# Microglial adenosine A_2A_ receptor in the paraventricular thalamic nucleus regulates pain sensation and analgesic effects independent of opioid and cannabinoid receptors

**DOI:** 10.3389/fphar.2024.1467305

**Published:** 2024-12-19

**Authors:** Yiping Cao, Zhou Wu, Moruo Zhang, Ran Ji, Hongxing Zhang, Lingzhen Song

**Affiliations:** ^1^ Jiangsu Province Key Laboratory of Anesthesiology, Xuzhou Medical University, Xuzhou, Jiangsu, China; ^2^ Jiangsu Province Key Laboratory of Anesthesia and Analgesia Application Technology, Xuzhou Medical University, Xuzhou, Jiangsu, China; ^3^ NMPA Key Laboratory for Research and Evaluation of Narcotic and Psychotropic Drugs, Xuzhou Medical University, Xuzhou, Jiangsu, China

**Keywords:** A_2A_ receptor, PVT, microglia, pain sensation, opioid receptor, cannabinoid receptor, non-opioid analgesia

## Abstract

**Introduction:**

The paraventricular thalamic nucleus (PVT) is recognized for its critical role in pain regulation, yet the precise molecular mechanisms involved remain poorly understood. Here, we demonstrated an essential role of the microglial adenosine A_2A_ receptor (A_2A_R) in the PVT in regulating pain sensation and non-opioid analgesia.

**Method and results:**

Specifically, A_2A_R was predominantly expressed in ionized calcium binding adapter molecule 1 (Iba1)-positive microglia cells within the PVT, with expression levels remaining unchanged in mice experiencing persistent inflammatory pain induced by complete Freund’s adjuvant (CFA). Pharmacological activation of local PVT A_2A_R with its agonist CGS21680 induced significantly decreased 50% paw withdrawal threshold (50%PWTs) and paw withdrawal latency (PWLs), as measured by the Von Frey test and Hargreaves test in adult mice. Conversely, intra-PVT infusion of A_2A_R antagonist SCH58261 increased 50%PWTs and PWLs in mice; a robust analgesic effect was also observed in CFA mice with inflammatory pain. Importantly, these analgesic effects of A_2A_R antagonist SCH58261 were not affected by adjunctive intraperitoneal administration of naloxone or rimonabant, inhibitors of opioid receptor and cannabinoid CB1 receptor (CB1R), respectively.

**Discussion:**

Overall, these pharmacological experiments underscore an essential role of microglia-expressed A_2A_R with in PVT in pain sensation while revealing a novel analgesic action independent of opioid and cannabinoids receptors. Thus, these findings highlight PVT microglial adenosine A_2A_ receptor as a promising target for novel approaches to pain modulation and future analgesic development.

## Introduction

Pain is a common and multifaceted clinical phenomenon, often indicative of underlying pathological processes and at times manifesting as a disorder itself. Historically, opioids have been pivotal in management of clinical pain; nevertheless, their use is frequently accompanied by a range of adverse effects including addiction, tolerance, and respiratory depression (Stein, 2018; Olson et al., 2017). The misuse of and addiction to opioids, including prescription pain relievers, heroin, and synthetic fentanyl, is now a severe crisis that impacts public health and socioeconomic welfare worldwide. This “Opioid Crisis” highlights the urgent necessity for new, safer analgesics. Given the challenges in developing side-effect-free opioid (Olson et al., 2017), there is an increasing interest in exploring new targets for pain regulation and non-opioid analgesia. However, the neural and molecular mechanisms underpinning non-opioid analgesia remain largely unexplored.

The paraventricular thalamic nucleus (PVT) functions as a critical relay nucleus and integrative hub within the complex neural network involved in behavioral regulation. Clinical and preclinical studies indicate that the PVT, predominantly composed of glutamatergic neurons, plays a critical role in pain modulation and associated abnormalities (Barson et al., 2020; Zhang et al., 2023; Zhu et al., 2016; Cui et al., 2024; Chang et al., 2019; Tang et al., 2023; Liang et al., 2020). For example, increased neuronal activity was noted in anterior PVT neurons in mice with neuropathic pain (Cui et al., 2024), while inhibition of PVT neurons has been shown to attenuate nociceptive hypersensitivity in mouse models of both neuropathic and visceral pain (Chang et al., 2019; Jurik et al., 2015). Chemogenetic or optogenetic activation of the GABAergic projection from the rostral zona incerta to the PVT resulted in alleviation of pain, while inhibiting this circuit led to mechanical hypersensitivity and partial heat hyperalgesia (Wu et al., 2023). Optogenetic manipulation of the PVT and its projections to the basolateral amygdala regulates both pain- and anxiety-like behavioral phenotypes (Tang et al., 2023). The posterior portion of the PVT and their downstream central amygdala→ventrolateral periaqueductal gray pathway are implicated in descending nociceptive facilitation regarding the development of neuropathic pain conditions in rats (Liang et al., 2020). Consistently, we recently demonstrated significantly elevated real-time calcium signals in the PVT following exposure to noxious thermal or mechanical stimuli (Zhang et al., 2023). At a neural circuitry level, excitability among PVT neurons projecting to the nucleus accumbens (PVT^glu^→NAc pathway) was enhanced during persistent pain induced by intraplantar injection of complete Freund’s adjuvant (CFA) (Zhang et al., 2023). As expected, repeated activation of the PVT projection towards the nucleus accumben (NAc) induced long-lasting pain-like behaviors; conversely, its inhibition reduced both intensity and duration of inflammatory pain experiences (Zhang et al., 2023). Collectively, these findings highlight the potential for targeting the PVT as a promising avenue for developing novel analgesic therapies aimed at alleviating various pain syndromes. However, further investigation into its underlying molecular targets remains necessary.

We recently conducted a profiling of molecule expression in PVT-NAc projecting glutamatergic neurons and identified dopamine receptor 3 and 5-hydroxytryptamine receptor 1D as promising targets for non-opioid analgesia, as well as for the treatment of chronic pain and depression comorbidity (Zhang et al., 2023; Cui et al., 2024). Notably, a robust expression of adenosine A_2A_ receptor (A_2A_R) was also detected in the PVT, but not in PVT-NAc projecting glutamatergic neurons (Zhang et al., 2023). Nevertheless, the distribution of A_2A_R in local cell types and its potential functional roles in pain regulation were not well known (Zhang et al., 2023).

In the present study, we aimed to elucidate the involvement of A_2A_R in PVT in pain behaviors by using selective A_2A_R agonists and antagonists. First, we identified a distinct expression pattern of A_2A_R exclusively within Iba1-positive microglia. Although A_2A_R expression was unchanged in the PVT following intraplantar CFA injection, intra-PVT infusion of an A_2A_R agonist induced pain-like behavior in adult mice, whereas an A_2A_R antagonist alleviated CFA-induced pain symptoms. Subsequent experiments showed that the analgesic effect remained consistent even with adjunctive administration of opioid and cannabinoid CB1 receptor (CB1R) antagonists. These results indicate a novel analgesic mechanism involving the modulation of adenosine signaling within the PVT as a potential pathway for pain relief.

## Materials and methods

### Animal and housing

All experiments were reviewed and approved by the Animal Care and Use Committee of Xuzhou Medical University (202207S034) and performed in accordance with the National Institutes of Health Guidelines and Use of Laboratory Animals and the Committee for Research and Ethical Issues of the International Association for the Study of Pain. Mice were group-housed (maximum five mice per cage) under a 12-h light/dark cycle (light from 8:00 a.m. to 8:00 p.m.), with food and water available *ad libitum*. The ambient temperature was maintained at 23°C ± 2°C with 55%–60% relative humidity. Only C57BL/6J male mice (8–14 weeks old) of normal appearance and weight (21 ± 2 g) were used for all studies. No food or water is available during modeling and testing (1–2 h). All mice were divided into different groups randomly. All tests were carried out during the light cycle between 9 a.m. and 4 p.m., and the investigators were blinded to experimental conditions during testing. Efforts were made to minimize animal suffering and reduce the number of animals used. Mice dropped out and be euthanized whose body-weight reduced 20% or more, or showed significant unhealthy condition after surgeries.

### Stereotactic surgeries

All surgeries were conducted under aseptic conditions. Mice were deeply anesthetized with an intraperitoneal injection of 1% sodium pentobarbital (40 mg/kg, i.p.) and placed in a stereotaxic frame (RWD Life Technology Co. LTD, Shenzhen, China). The eyes of the mice were kept moist using ophthalmic ointment throughout the surgery. The skull plane was adjusted to ensure that the bregma and lambda were at a horizontal level. Small holes were drilled in the skull above the target brain region using a dental drill to lower a unilateral cannula (RWD Life Technology Co, Ltd.) into the PVT (AP = −0.85 mm, ML = ±0.05 mm, DV = −3.45 mm). Once the cannula is positioned correctly, bone cement (usually a fast setting polymethyl methacrylate) is prepared. The cement is carefully applied around the base of the cannula where it meets the skull. Care must be taken to avoid the cannula and ensure that the cement only contacts the outer surface of the skull and cannula holder. A stainless-steel obturator was inserted into each guide cannula to prevent blockage. After the operation, mice were placed in a cage with a heating pad to keep warm and returned to their home cage when fully awake. Mice were allowed to recover for at least 1 week before the behavioral experiments.

### Intra-PVT drug infusions

The effects of different adenosine agonists and antagonist on nociceptive behavior were assessed. A_2A_Rs agonist CGS21680 hydrochloride (MedChemExpress), A_2A_Rs antagonist SCH58261 MedChemExpress) were firstly dissolved in DMSO, then diluted with 0.9% saline. A volume of 1 μL of vehicle (0.1% DMSO) or drug was injected at the rate of 0.2 μL/min. The injector cannula was held for 5 min before withdrawal to minimize drug spread along the injection track. Behavior tests were conducted after 30 min, and within 90 min after the drug infusion. To examine whether the effect of SCH58261 on pain behavior depends on opioid and cannabinoid CB1 receptor (CB1R), naloxone (2 mg/kg) or rimonabant (5 mg/kg) was administrated intraperitoneally 30 min before the intra-PVT drug infusion. In the CFA pain model, drug infusions were given (single dose) at 4 h or 3 days following CFA injection, when mechanical allodynia was fully developed. Morphine (5 mg/kg) was administrated intraperitoneally 30 min after naloxone, and pain threshold were measured at least 30 min after injection of morphine. Throughout all behavioral experiments, the observer was blinded to the treatment.

### Complete freund’s adjuvants model

Completed Freund’s Adjuvants (CFA, Beyotime, P2036) were used to establish the persistent inflammatory pain model. 15 μL of CFA was unilaterally injected into the intra-plantar surface of the left hind paw with a 20-gauge micro-injector. Mice from the Sham group received the same volume of the Saline instead. Four hours and 3 days following the injection, behavioral tests were performed to evaluate the acute and persistent pain.

### Von frey test and 50% paw withdrawal threshold (50%PWT)

Responses to mechanical stimulation by von Frey hairs (von Frey filaments; Stoelting, Kiel, WI, United States) (0.008, 0.02, 0.07, 0.16, 0.4, 0.6, 1, 2, and 6 g) were determined in the plantar surface of the left hind paw. Positive responses included licking, biting, shaking, and sudden withdrawal of the hind paws; otherwise, negative. Mechanical thresholds were tested using the up-down method. Stimuli were always presented consecutively, whether ascending or descending. Starting from 0.16 g, in the absence of a positive response to the initially selected filament, a stronger stimulus was presented; in the event of a positive response, the next weaker stimulus was chosen until the first positive and negative was crossed, and then measured 4 times in a row (more than 30 s between each stimulus). The resulting pattern of positive and negative responses was tabulated using the convention, X = positive response; O = negative response, and the response threshold was interpolated using the formula: 50%PWT = 10 [log(X)+κδ], where *X*_f_ = value (in log units) of the final von Frey filament used; k = the coefficient of different stimuli for the pattern of positive/negative responses; and δ = mean difference (in log units) between stimuli (here, 0.410723). Mice were pre-acclimated to the testing environment for 2 days before baseline testing and then placed individually under inverted clear plexiglass boxes (length: 8 cm × width: 8 cm × height: 5.5 cm split into four quadrants) on an elevated metal mesh rack and allowed to habituate for at least 40 min before each testing. All nociceptive sensitizations described here are of relatively short duration (1–10 min) and allow the mouse to withdraw its paw from the painful stimulus. All tests were performed in a blinded manner.

### Hargreaves test and paw withdrawal latency (PWL)

The mice are first acclimatized to the testing environment. They are placed individually under inverted clear plexiglass boxes (length: 8 cm × width: 8 cm × height: 5.5 cm split into four quadrants) on an elevated glass platform. The mice are allowed to habituate for at least 30 min to minimize stress and ensure consistent baseline measurements. Once the mice are acclimatized, they are positioned so that one of their hind paws rests flat on the glass surface. A focused beam of light is directed at the plantar surface of the left hind paw. The intensity of the heat stimulus is calibrated to avoid tissue damage while being sufficiently noxious to elicit a withdrawal response. The time from the onset of the heat stimulus to the withdrawal of the hind paw is recorded as the paw withdrawal latency (PWLs). This latency is an indicator of the mouse’s pain threshold. To avoid tissue damage and ensure humane treatment, a cutoff time (20 s) is set, after which the heat source is automatically turned off if no withdrawal response is observed. The test is typically repeated multiple times with sufficient intervals between trials to prevent sensitization or habituation. The baseline measurements are taken before any experimental treatments are applied. The withdrawal latencies are averaged for each mouse, and comparisons are made between control and treatment groups to assess the effects of experimental interventions on thermal pain sensitivity.

### Immunohistochemistry

Mice were deeply anesthetized with intraperitoneal injection of 1% sodium pentobarbital (40 mg/kg, i.p.) and transcardially perfused with 40 mL of 0.01 M cold phosphate-buffered saline (PBS, pH = 7.4), followed by 20 mL of PBS containing 4% paraformaldehyde. Immediately after perfusion, the brains were carefully removed and post-fixed in 4% paraformaldehyde at 4°C for 24 h, then transferred to a 20% sucrose-PBS solution for another 24 h, followed by 30% sucrose for an additional 24 h for cryoprotection. Coronal sections (30 μm) were prepared using a cryostat microtome (Leica VT1000S, Germany). For immunohistochemistry, these sections were rinsed in PBS three times for 5 min each, followed by blocking of non-specific reactions with 1% bovine serum albumin in PBS containing 0.4% Triton X-100 for 45 min at room temperature. Subsequently, sections were incubated overnight at 4°C with appropriate primary antibodies diluted in TBS on a shaker. The primary antibodies used were: mouse anti-A_2A_R (1:50, Santa Cruz, sc-32261), rabbit anti-Iba1 (1:500, Wako, PRT2404), rabbit anti-GFAP (1:500, Proteintech, 16825-1-AP), and rabbit anti-NeuN (1:500, Proteintech, 26975-1-AP). After overnight incubation, brain sections were washed three times in TBS for 10 min each and then incubated for 2 h with the corresponding fluorophore-conjugated secondary antibodies: anti-mouse Alexa 488 (1:500, Thermo Fisher Scientific, A28175) and anti-rabbit Alexa 594 (1:500, Thermo Fisher Scientific, A11012), diluted in TBS. After secondary antibody incubation, sections were washed twice in TBS for 10 min each, followed by one wash in PBS for 10 min at room temperature. Finally, sections containing the PVT were mounted on glass slides with Fluoroshield containing DAPI (Abcam, ab104139) and coverslipped. Images were acquired using a confocal laser-scanning microscope (Leica STELLARIS 5, Germany; FV1000, Olympus, Japan).

### Western blotting

PVT brain tissue was harvested and sonicated in 200 μL lysis buffer containing phosphatase and protease inhibitors. After centrifugation (12,000 rpm, 15 min), the supernatants were collected for protein concentration assay using an OD-assay facility (Thermo Scientific, United States). Proteins (40 μg per lane) were separated by electrophoresis on a 10% SDS-PAGE gel and transferred onto PVDF membranes (Millipore, United States). The membranes were rinsed and blocked in a 3% BSA solution (Sigma-Aldrich, V900933) for 2 h at room temperature, followed by overnight incubation at 4°C with primary antibodies: rabbit anti-GAPDH (1:1,000, Proteintech, 10494-1-AP) and mouse anti-A_2A_R (1:50, Santa Cruz, sc-32261). The membranes were then washed and incubated with a secondary antibody conjugated to alkaline phosphatase (1:1,000, Vicmed, VA006) for 2 h at room temperature. Finally, protein bands were visualized using a coloring solution containing BCIP (Promega, S381C) and NBT (Promega, S380C). Grayscale analysis of the A_2A_R protein bands was performed and standardized against GAPDH.

### Statistics

Data from mice with missed injections were excluded from further analysis by experimenters who were blinded to the experimental conditions. The normality of data was assessed using the Shapiro-Wilk test. Non-normally distributed data were compared using nonparametric tests (Mann-Whitney U test for comparing data between two groups, while the Kruskal–Wallis test was applied for comparisons involving multiple groups). Normally distributed data with equal variance were analyzed using unpaired two-tailed t-tests. For multiple comparisons, one-way analysis of variance (ANOVA) was employed, followed by Tukey’s *post hoc* test for determining statistical significance. All parametric data are presented as mean ± SEM, nonparametric data as medians with interquartile ranges. Statistical significance levels were denoted as *p < 0.05, **p < 0.01, ***p < 0.001. GraphPad Prism 9 (GraphPad Software, Inc.) and Adobe Illustrator was used for statistical analyses and graphing purposes.

## Results

### Cell type-specific expression of A_2A_R in the PVT

**FIGURE 1:**
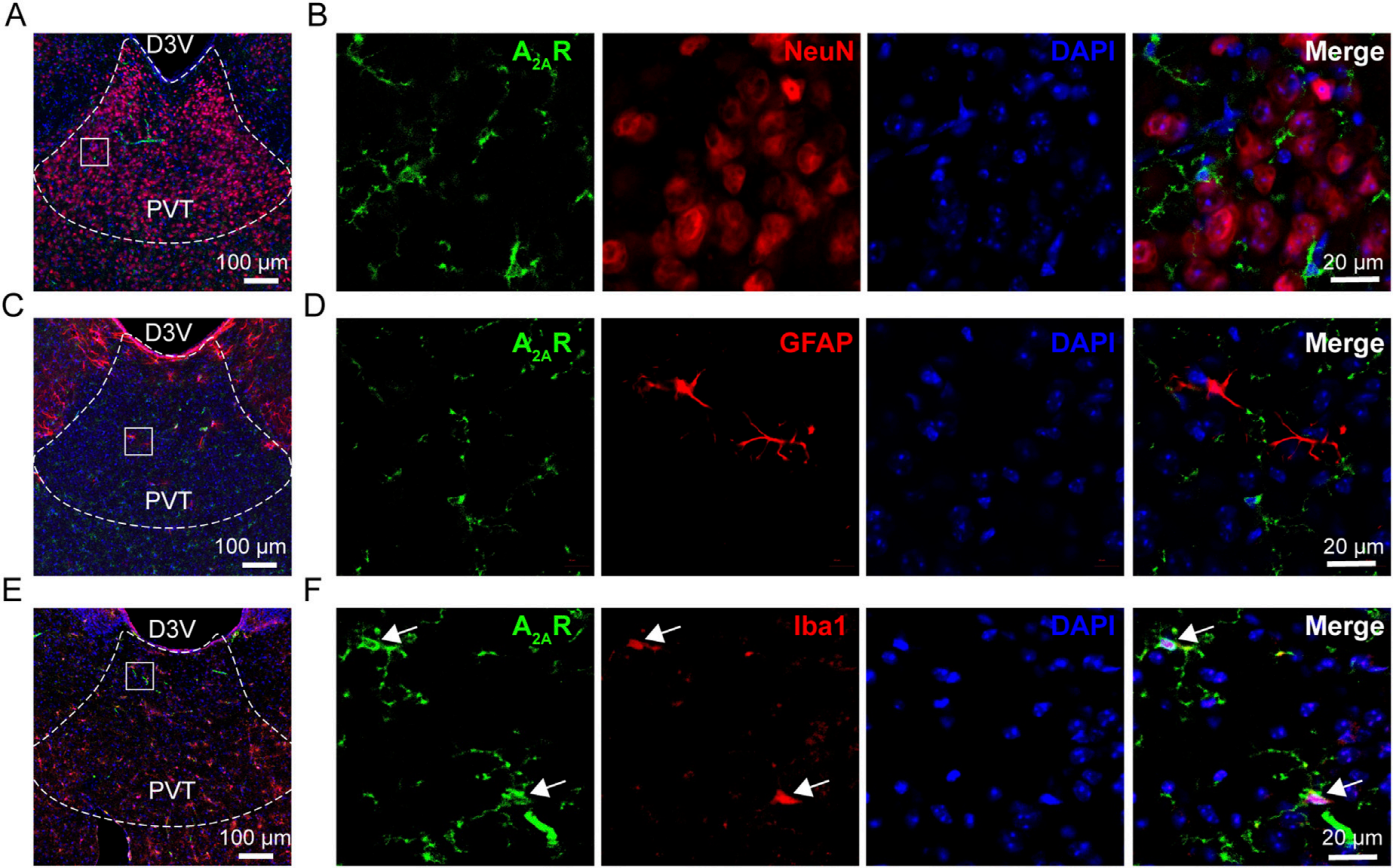
A_2A_R expression in the PVT. **(A, B)** Representative immunofluorescent images showing the expression of A_2A_R (green) and staining of NeuN for neurons (red). **(C, D)** Representative immunofluorescent images showing the expression of A_2A_R (green) and staining of GFAP for astrocytes (red). **(E, F)** Representative immunofluorescent images showing the expression of A_2A_R (green) and staining of Iba1for microglia (red). Scale bar = 100 µm in **(A, C, E)**, scale bar = 20 µm in **(B, D, F)**.

Previous studies have demonstrated the expression of A_2A_R in the brain (Wang et al., 2022; Cen et al., 2023), as well as in paraventricular thalamic nucleus (PVT) (Zhang et al., 2023). Here, we conducted immunofluorescent staining to further examine the distribution of A_2A_R in different cell types. To do this, anti-A_2A_R staining was performed with neuron marker NeuN, which corresponding to more than 90% cells in PVT. Our result indicated that A_2A_R is not expressed in PVT neurons (Figures 1A, B). Subsequently, we performed similar staining with astrocyte marker glial fibrillary acidic protein (GFAP, Figures 1C, D) and microglia marker ionized calcium-binding adapter molecule (Iba1, Figures 1E, F), respectively. Our results demonstrated an overlap of A_2A_R staining with Iba1, but not with GFAP. Together, these data demonstrate a cell type-specific expression of A_2A_R within PVT microglia, but not in neuron or astrocyte.

### CFA inflammatory pain did not affect the expression of PVT A_2A_R

**FIGURE 2:**
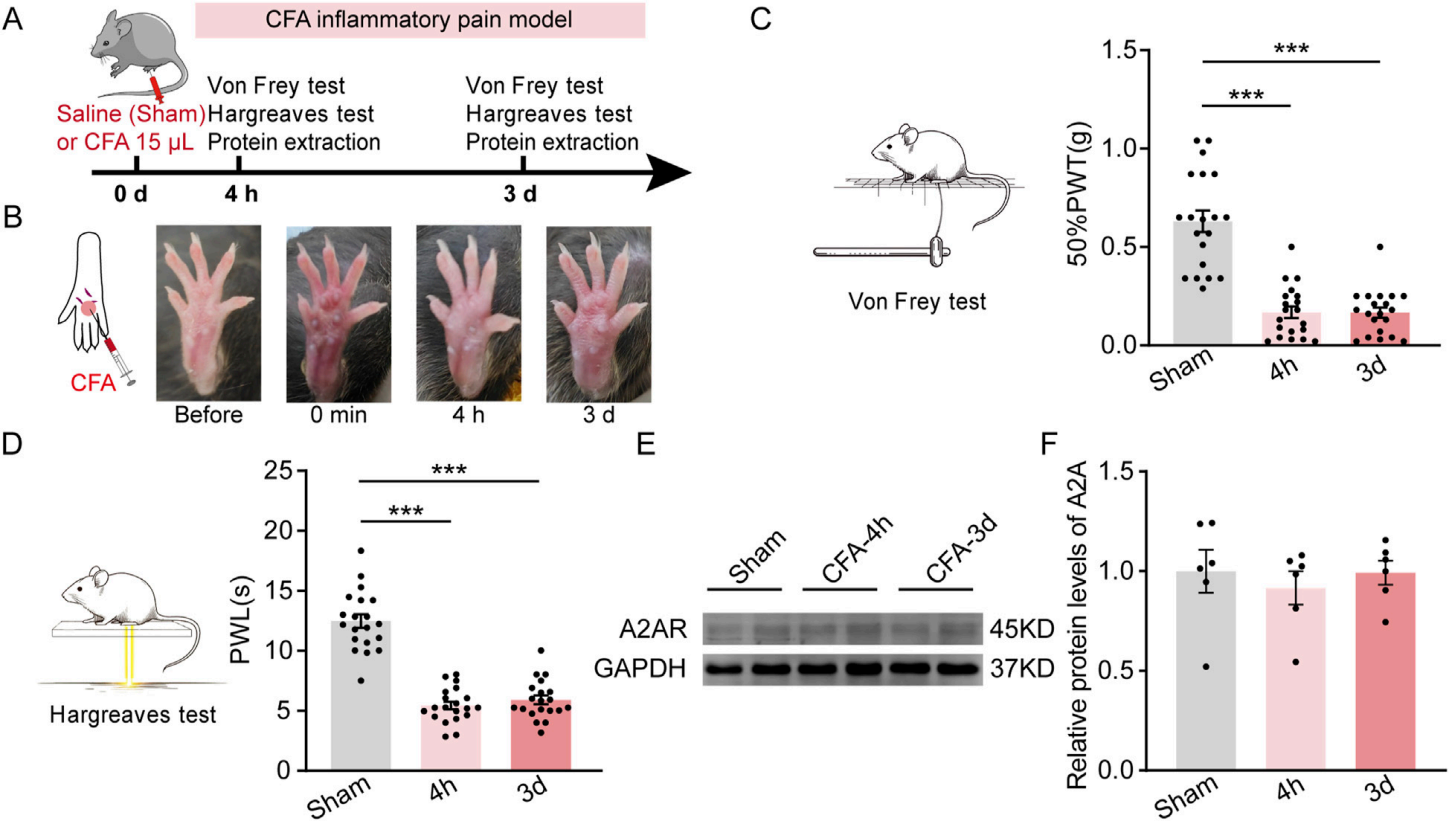
CFA induced inflammatory pain does not affect PVT A_2A_R expression. **(A)** An experimental timeline for CFA modeling, Von Frey and Hargreaves tests, and protein detection at specified time points. **(B)** Representative images showing the CFA affected hindpaw before and after injection. **(C)** Von Frey test results show significantly decreased mechanical pain threshold (50%PWTs) in CFA-treated mice at 4 h (CFA-4h) and 3 days (CFA-3d) timepoints. (Sham, *n* = 20 mice; 4 h, *n* = 20 mice; 3 days, *n* = 20 mice). **(D)** Hargreaves test results show significantly shortened thermal pain latency (PWLs) in CFA-treated mice at 4 h (CFA-4h) and 3 days (CFA-3d) after injection. (Sham, n = 20 mice; 4 h, n = 20 mice; 3 days, n = 20 mice). **(E, F)** Representative western blots and quantitative data of A_2A_R protein expression in mice received saline and CFA treatments. (Sham, *n* = 6 mice; 4 h, *n* = 6 mice; 3 days, *n* = 6 mice). ***p < 0.001. Data analyzed with **(C, D, F)** one-way ANOVA followed by Tukey post-tests. Error bars indicate SEM.

Next, we examined the expression of PVT A_2A_R in mice experiencing persistent inflammatory pain induced by an intra-plantar injection of complete Freund’s adjuvant (CFA) (Figure 2A). Significant local edema and redness was visually observed in the affected hindpaw of CFA mice, indicating a state of inflammatory, which persisted for at least 3 days (Figure 2B). Consistent with our previous studies, CFA mice displayed decreased 50% paw withdrawal threshold (50%PWTs) and paw withdrawal latency (PWLs) when tested 4 h and 3 days after CFA injection (Figures 2C, D; Supplementary Table 1) (Figure 2C: Sham, *n* = 20 mice; 4 h, *n* = 20 mice; 3 days, *n* = 20 mice, F (2, 57) = 50.19, *p* < 0.0001, One-way ANOVA with Tukey’s *post hoc* tests; Figure 2D: Sham, *n* = 20 mice; 4 h, *n* = 20 mice; 3 days, *n* = 20 mice, F (2, 57) = 84.89, *p* < 0.0001, One-way ANOVA with Tukey’s *post hoc* tests). Unexpectedly, no significant changes were detected in PVT A_2A_R protein expression level in CFA-treated mice (Figures 2E, F; Supplementary Figure 1; Supplementary Table 1) (Sham, *n* = 6 mice; 4 h, *n* = 6 mice; 3 days, *n* = 6 mice, F (2, 15) = 0.2905, *p* = 0.7734, One-way ANOVA with Tukey’s *post hoc* tests). These results suggest CFA induced inflammatory pain does not affect the expression of PVT A_2A_R, and imply that if A_2A_R plays any role in mediating the persistent inflammatory pain state, it might not by changes in its protein expression.

### Pharmacological manipulation of PVT A_2A_R activity bi-directionally regulates pain behaviors

**FIGURE 3:**
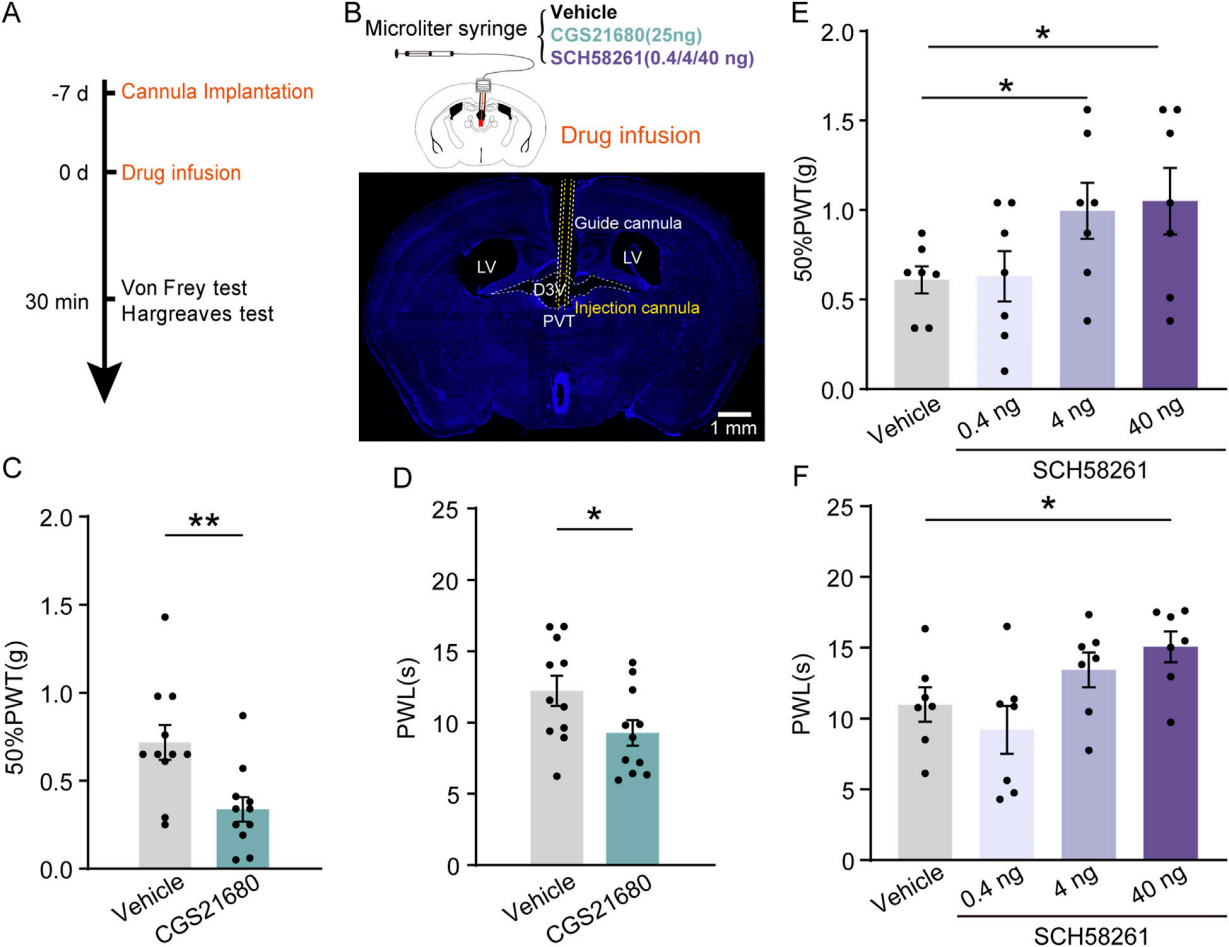
Pharmacological manipulation of PVT A_2A_R regulates pain behaviors in mice. **(A)** Experimental timeline for cannula implantation, drug infusion (CGS21680 and SCH58261), and Von Frey and Hargreaves tests. **(B)** Diagram of injection cannula in the PVT region. **(C, D)** 50%PWTs **(C)** and PWLs **(D)** in mice treated with vehicle and CGS21680 (Vehicle, *n* = 11 mice; CGS21680, *n* = 11 mice). **(E, F)** 50%PWTs **(E)** and PWLs **(F)** in mice treated with vehicle and SCH58261. (Vehicle, *n* = 11 mice; 0.4 ng, *n* = 11 mice; 4 ng, *n* = 11 mice; 40 ng, *n* = 11 mice). *p < 0.05, **p < 0.01. Data analyzed with unpaired *t*-test **(C, D)**, or one-way ANOVA followed by Tukey post-tests **(E, F)**. Error bars indicate SEM. PVT, paraventricular nucleus. D3V, Third ventricle. LV, Lateral ventricles.

Then we investigated whether pharmacological manipulation of local A_2A_R activity plays a role in regulating pain sensation in both control (CON) and CFA mice. A unilateral cannula was implanted into the PVT for local administration of either A_2A_Rs agonists or its antagonists, 30 min before behavioral tests (Figures 3A, B). Our behavioral tests revealed that a single infusion of A_2A_R agonists CGS21680 [24 μg/μL in 1 μL (Ferré et al., 2023)] significantly decreased both the 50%PWTs (Figure 3C; Supplementary Table 1) [Vehicle, n = 11 mice, CGS21680, n = 11 mice, t (20) = 3.139, *p* = 0.0052, Unpaired *t*-test] and PWLs (Figure 3D; Supplementary Table 1) [Vehicle, n = 11 mice, CGS21680, n = 11 mice, t (20) = 2.213, *p* = 0.0465, Unpaired *t*-test] in Sham mice under physiological state. To test the effect of A_2A_R antagonism on the behavioral outcome, we infused 3 different dose of its antagonist SCH58261 into the PVT and found that a low dose of SCH58261 (0.4 ng/μL in 1 μL) had no effect on either the 50%PWTs (Figure 3E; Supplementary Table 1) (Vehicle, *n* = 7 mice; SCH58261 0.4 ng, n = 7 mice, SCH58261 4 ng, n = 7 mice, SCH58261 40 ng, n = 7 mice, F (3, 24) = 4.163, *p* = 0.0165, One-way ANOVA with Tukey’s *post hoc* test) or PWLs (Figure 3F; Supplementary Table 1) (Vehicle, *n* = 7 mice; SCH58261 0.4 ng, n = 7 mice, SCH58261 4 ng, n = 7 mice, SCH58261 40ng, n = 7 mice, F (3, 24) = 4.192, *p* = 0.0161, One-way ANOVA with Tukey’s *post hoc* test), while a high dose (40 ng/μL in 1 μL) increased both 50%PWTs (Figure 3E) and PWLs (Figure 3F). Moreover, a medium dose of SCH58261 (4 ng/μL in 1 μL) was sufficient to increase the 50%PWTs but not the PWLs in CON mice (Figures 3E, F). These data suggest a dose-depended analgesic role of A_2A_Rs antagonist SCH58261 when infused into the PVT. Locomotion of the animals after PVT infusion of A_2A_R antagonist SCH58261 (40 ng) were examined in an open filed arena (50 cm*50 cm). 30 min after intra-fusion of SCH58261, their movement was recorded and the total moved distance after SCH58261 treatment was significant higher than Vehicle-treated mice (Supplementary Figure 2; Supplementary Table 1, Vehicle, *n* = 8 mice, SCH, *n* = 7 mice), indicating that intra-PVT infusion of SCH58261 is not anesthetic, but rather analgesic. Together, the pharmacological evidence presented above indicates a bi-directional regulatory effect on pain behaviors by targeting local A_2A_Rs within the PVT.

**FIGURE 4:**
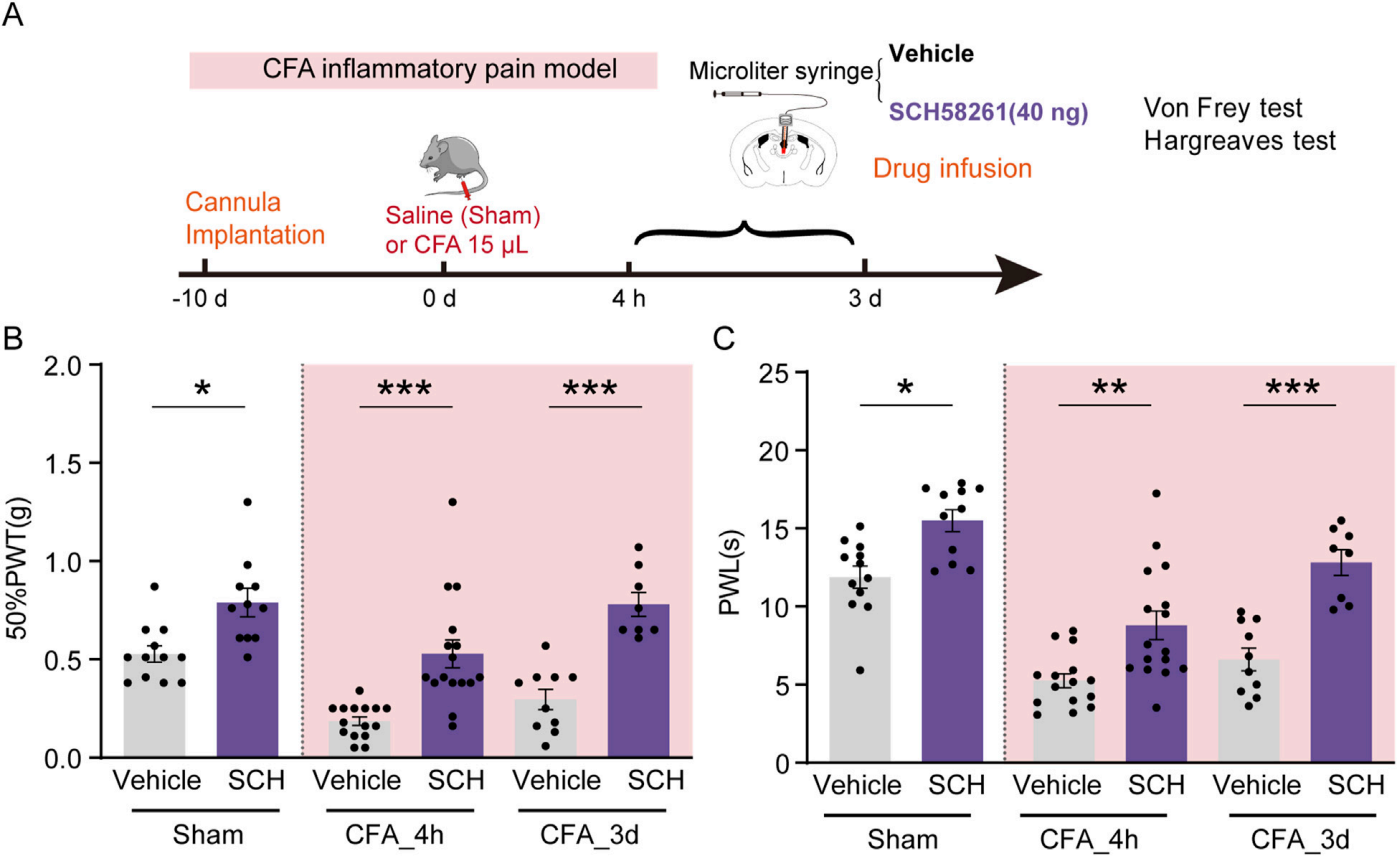
PVT A_2A_R antagonism alleviated CFA inflammatory pain. **(A)** Experimental timeline for cannula implantation, CFA or Saline (Sham) injection, drug infusion (SCH58261), and Von Frey and Hargreaves tests. **(B, C)** 50%PWTs and PWLs in Sham and CFA mice received vehicle or SCH58261 treatment at different time points following CFA injection. Von Frey test results show significantly increased mechanical pain threshold in 40 ng SCH58261-treated group compared to Vehicle group. (Sham Vehicle, *n* = 12 mice; Sham SCH, *n* = 11 mice; CFA_4h Vehicle, *n* = 15 mice; CFA_4h SCH, *n* = 16 mice; CFA_3d Vehicle, *n* = 10 mice; CFA_3d SCH, *n* = 8 mice). *p < 0.05, **p < 0.01, ***p < 0.001. Data analyzed by **(B, C)** two-way ANOVA with Tukey post-tests. Error bars indicate SEM.

To further validate the analgesic effect of PVT A_2A_Rs inhibition in CFA mice, SCH58261 (40 ng/μL in 1 μL) was infused into the PVT 30 min before behavioral tests (Figure 4A). Intraplantar injection of CFA successfully reduced the pain threshold compared to Saline-injected Sham group. Our results consistently demonstrated that pretreatment with SCH58261 remarkably increased the 50%PWTs and PWLs in sham and CFA mice (Figures 4B, C; Supplementary Table 1) (Figure 4B: Sham Vehicle, *n* = 12 mice, Sham SCH, *n* = 11 mice, CFA_4h Vehicle, *n* = 15 mice, CFA_4h SCH, *n* = 16 mice, CFA_3d Vehicle, *n* = 10 mice, CFA_3d SCH, *n* = 8 mice, F (5, 66) = 18.33, *p* < 0.0001, One-way ANOVA with Tukey’s *post hoc* test; Figure 4C: Sham Vehicle, *n* = 12 mice, Sham SCH, *n* = 11 mice, CFA_4h Vehicle, *n* = 15 mice, CFA_4h SCH, *n* = 16 mice, CFA_3d Vehicle, *n* = 10 mice, CFA_3d SCH, *n* = 8 mice, F (5, 66) = 26.78, *p* < 0.0001, One-way ANOVA with Tukey’s *post hoc* test).

Taken together, these results indicate that pharmacological activation of PVT A_2A_R is sufficient to elicit pain-like behaviors while inhibition of this receptor displays robust analgesic effects in both Sham and CFA mice.

### A_2A_R antagonism induced analgesic effects did not require opioid receptors

**FIGURE 5:**
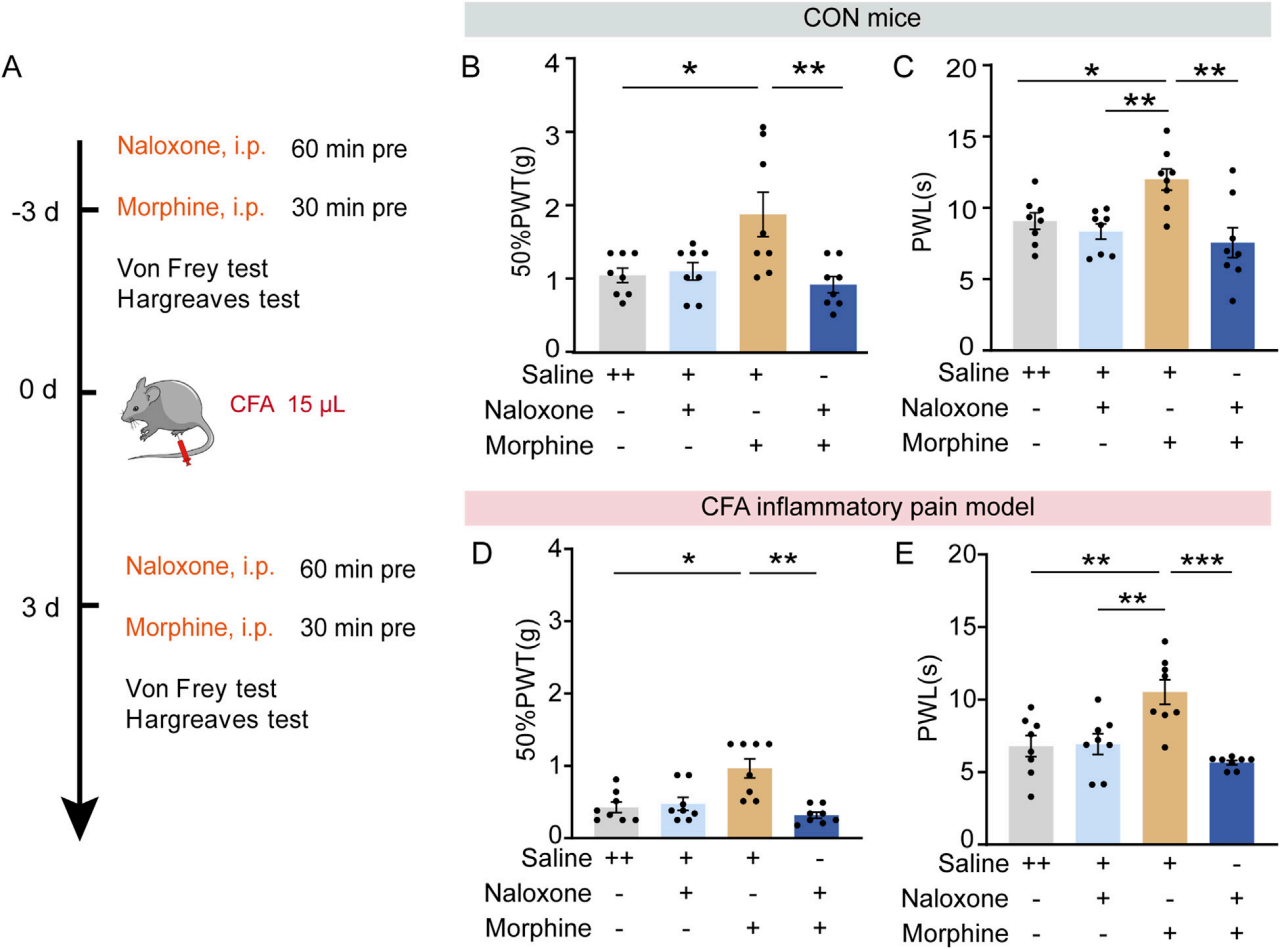
Opioid dependent analgesic effects of morphine. **(A)** Experimental timeline for pharmacological treatments, CFA injectionand behavioral tests. **(B)** Von Frey test results show significantly increased 50%PWTs in morphine treated mice which can be prevented by an adjunctive injection of naloxone. **(C)** Hargreaves test results show significantly increased PWLs in morphine treated mice which can be prevented by an adjunctive injection of naloxone. (Saline + Saline, *n* = 8 mice; Saline + Morphine, *n* = 8 mice; Naloxone + Saline, *n* = 8 mice; Naloxone + Morphine, *n* = 8 mice). **(D)** Von Frey test results show significantly increased 50%PWTs in morphine treated CFA mice, which can be prevented by an adjunctive injection of naloxone. **(E)** Hargreaves test results show significantly increased PWLs in morphine treated CFA mice, which can be prevented by an adjunctive injection of naloxone. (Saline + Saline, *n* = 8 mice; Saline + Morphine, *n* = 8 mice; Naloxone + Saline, *n* = 8 mice; Naloxone + Morphine, *n* = 8 mice). *p < 0.05, **p < 0.01, ***p < 0.001. Data **(B, C, E)** analyzed with one-way ANOVA followed by Tukey post-tests; Data **(D)** analyzed with one-way ANOVA followed by Kruskal–Wallis test. Error bars indicate SEM.

Intra-PVT infusion significantly elevated the PWLs and 50%PWTs in control mice (Figures 3E, F; Figures 4B, C), an analgesia-like behavioral effect that was also observed in morphine-treated mice (5 mg/kg *i.p.*, 30 min before the behavioral test, Figures 5A–C; Supplementary Table 1) (Figure 5B: Saline + Saline, *n* = 8 mice, Saline + Morphine, *n* = 8 mice, Naloxone + Saline, *n* = 8 mice, Naloxone + Morphine, *n* = 8 mice, F (3, 28) = 5.896, *p* = 0.0030, One-way ANOVA with Tukey’s *post hoc* test; Figure 5C: Saline + Saline, *n* = 8 mice, Saline + Morphine, *n* = 8 mice, Naloxone + Saline, *n* = 8 mice, Naloxone + Morphine, *n* = 8 mice, F (3, 28) = 6.575, *p* = 0.0017, One-way ANOVA with Tukey’s *post hoc* test). As expected, analgesic effects induced by morphine in CFA mice can be prevented by pretreatment with the opioid receptor antagonist naloxone (2 mg/Kg, *i.p.*, Figures 5D, E; Supplementary Table 1) (Figure 5D: Saline + Saline, *n* = 8 mice, Saline + Morphine, *n* = 8 mice, Naloxone + Saline, *n* = 8 mice, Naloxone + Morphine, *n* = 8 mice, H = 15.04, *p* = 0.0018, One-way ANOVA with Kruskal–Wallis test; Figure 5E: Saline + Saline, *n* = 8 mice, Saline + Morphine, *n* = 8 mice, Naloxone + Saline, *n* = 8 mice, Naloxone + Morphine, *n* = 8 mice, F (3, 28) = 10.06, *p* = 0.0001, One-way ANOVA with Tukey’s *post hoc* test). These results indicate an opioid receptor-dependent analgesic property of morphine. Previous studies has confirmed that interplay between opioid receptors, adenosine and A_2A_R in the dorsal horn of the spinal cord contribute to spinal analgesia (Sweeney et al., 1987). Whether the analgesic effect of SCH58261 is indirectly depend on the endogenous opioid systems was examined in our study.

**FIGURE 6:**
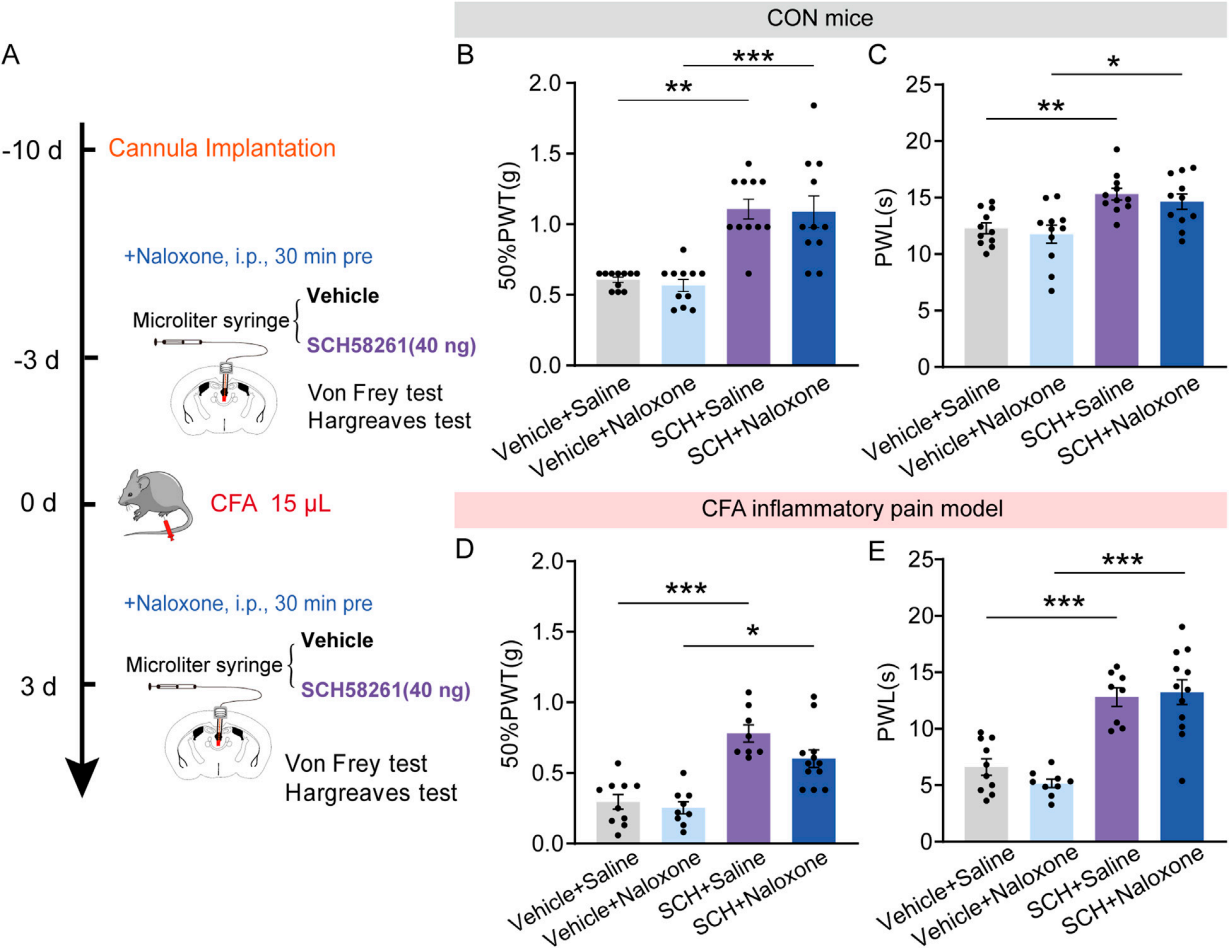
Intra-PVT infusion of A_2A_R antagonist induced analgesic effect that does not require opioid receptors. **(A)** Experimental timeline for pharmacological treatments, behavioral tests and CFA injection. **(B)** Von Frey test results show significantly increased 50%PWTs in SCH treated control (CON) mice, which cannot be prevented by naloxone treatment. **(C)** Hargreaves test results show significantly increased PWLs in SCH treated CON mice, which cannot be prevented by naloxone treatment. (Vehicle + Saline, *n* = 11 mice; Vehicle + Naloxone, *n* = 11 mice; SCH + Saline, *n* = 11 mice; SCH + Naloxone, *n* = 11 mice). **(D)** Von Frey test results show significantly increased 50%PWTs in SCH treated CFA mice, which cannot be prevented by naloxone treatment. **(E)** Hargreaves test results show significantly increased PWLs in SCH treated CFA mice, which cannot be prevented by naloxone treatment. (Vehicle + Saline, *n* = 10 mice; Vehicle + Naloxone, *n* = 9 mice; SCH + Saline, *n* = 8 mice; SCH + Naloxone, *n* = 12 mice). *p < 0.05, *p < 0.05, **p < 0.01, ***p < 0.001. Data **(B, D)** analyzed with one-way ANOVA followed by Kruskal–Wallis test; Data **(C, E)** analyzed with one-way ANOVA followed by Tukey post-tests. Error bars indicate SEM.

To determine the possible role of opioid receptors in the analgesic effect induced by SCH58261, naloxone was administrated intraperitoneally 30 min before intra-PVT infusion of SCH58261 (Figure 6A). Consistently, behavioral tests demonstrated a significant increase in 50%PWTs and PWLs in SCH58261 treated mice. Furthermore, SCH58251 induced analgesic effect cannot be prevented by naloxone treatment (Figures 6B, C; Supplementary Table 1) (Figure 6B: Vehicle + Saline, n = 11 mice, Vehicle + Naloxone, n = 11 mice, SCH + Saline, n = 11 mice, SCH + Naloxone, n = 11 mice, H = 28.55, *p* < 0.0001, One-way ANOVA with Kruskal–Wallis test; Figure 6C: Vehicle + Saline, n = 11 mice, Vehicle + Naloxone, n = 11 mice, SCH + Saline, n = 11 mice, SCH + Naloxone, n = 11 mice, F (3, 40) = 10.14, *p* < 0.0001, One-way ANOVA with Tukey’s *post hoc* test). Meanwhile, the same treatments were repeated in CFA inflammatory pain model, which showed significant reduced mechanical and thermal pain threshold. Similarly, SCH58251 induced an analgesic effect in CFA mice, which cannot be prevented by naloxone treatment (Figures 6D, E; Supplementary Table 1) (Figure 6D: Vehicle + Saline, n = 10 mice, Vehicle + Naloxone, n = 9 mice, SCH + Saline, n = 8 mice, SCH + Naloxone, n = 12 mice, H = 25.67, *p* < 0.0001, One-way ANOVA with Kruskal–Wallis test; Figure 6E: Vehicle + Saline, n = 10 mice, Vehicle + Naloxone, n = 9 mice, SCH + Saline, n = 8 mice, SCH + Naloxone, n = 12 mice, F (3, 35) = 23.43, *p* < 0.0001, One-way ANOVA with Tukey’s *post hoc* test).

These data indicate that intra-PVT infusion of SCH58261 induced analgesic action does not require opioid receptors.

### A_2A_R antagonism induced analgesic effects did not require cannabinoid receptor

**FIGURE 7:**
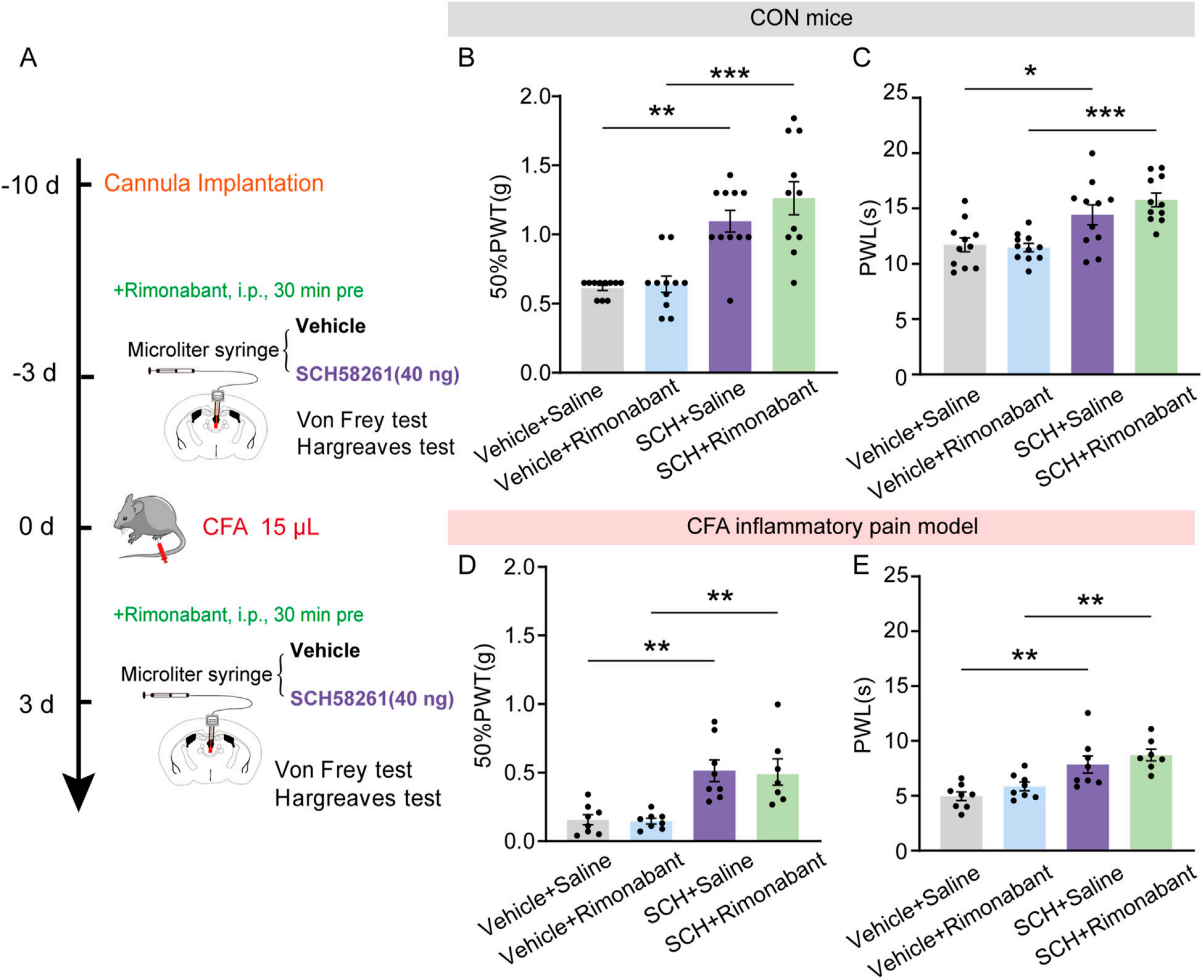
Intra-PVT infusion of A_2A_R antagonist induced analgesic effect that does not require cannabinoid receptors. **(A)** Experimental timeline for pharmacological treatments, behavioral tests and CFA injection. **(B)** Von Frey test results show significantly increased 50%PWTs in SCH treated control (CON) mice, which cannot be prevented by rimonabant treatment. **(C)** Hargreaves test results show significantly increased PWLs in SCH treated CON mice, which cannot be prevented by rimonabant treatment. (Vehicle + Saline, *n* = 11 mice; Vehicle + Rimonabant, *n* = 11 mice; SCH + Saline, *n* = 11 mice; SCH + Rimonabant, *n* = 11 mice). **(D)** Von Frey test results show significantly increased 50%PWTs in SCH treated CFA mice, which cannot be prevented by rimonabant treatment. **(E)** Hargreaves test results show significantly increased PWLs in SCH treated CFA mice, which cannot be prevented by rimonabant treatment. (Vehicle + Saline, *n* = 8 mice; Vehicle + Rimonabant, *n* = 8 mice; SCH + Saline, *n* = 8 mice; SCH + Rimonabant, *n* = 7 mice). *p < 0.05, **p < 0.01, ***p < 0.001. Data **(B)** analyzed with one-way ANOVA followed by Kruskal–Wallis test; Data **(C, D, E)** analyzed with one-way ANOVA followed by Tukey post-tests. Error bars indicate SEM.

Aside from the potential involvement of endogenous opioid systems in pain relief, the endocannabinoid system is now known as one of the key endogenous systems regulating pain sensation (Woodhams et al., 2015), primarily through its action on cannabinoid receptors. Next, we examine the potential role of cannabinoid receptor in mediating the non-opioid analgesic effect of A_2A_R antagonists. To achieve this goal, the aforementioned pharmacological experiment was repeated by replacing naloxone with a selective central CB1R antagonist rimonabant (5 mg/kg, *i.p.*, Figure 7A). Rimonabant failed to reverse the increase in 50%PWTs and PWLs induced by intra-PVT infusion of SCH58261 in CON mice (Figures 7B, C; Supplementary Table 1) (Figure 7B: Vehicle + Saline, n = 11 mice; Vehicle + Rimonabant, n = 11 mice; SCH + Saline, n = 11 mice; SCH + Rimonabant, n = 11 mice, H = 25.12, *p* < 0.0001, One-way ANOVA with Kruskal–Wallis test; Figure 7C: Vehicle + Saline, n = 8 mice; Vehicle + Rimonabant, n = 8 mice; SCH + Saline, n = 8 mice; SCH + Rimonabant, F (3, 27) = 9.611, *p* = 0.0002, One-way ANOVA with Tukey’s *post hoc* test), as well as in CFA inflammatory pain mice (Figures 7D, E; Supplementary Table 1) (Figure 7D: Vehicle + Saline, n = 8 mice; Vehicle + Rimonabant, n = 8 mice; SCH + Saline, n = 8 mice; SCH + Rimonabant, n = 7 mice, F (3, 27) = 10.29, *p* = 0.0001, One-way ANOVA with Tukey’s *post hoc* test; Figure 7E: Vehicle + Saline, n = 8 mice; Vehicle + Rimonabant, n = 8 mice; SCH + Saline, n = 8 mice; SCH + Rimonabant, F (3, 27) = 9.611, *p* = 0.0002, One-way ANOVA with Tukey’s *post hoc* test). This data further demonstrates that the analgesic effect of SCH58261 does not rely on the cannabinoid system.

Taken together, these data establish an essential role of microglial adenosine A_2A_ receptor within the paraventricular thalamic nucleus in regulating pain sensation and highlight an analgesic effect of PVT A_2A_R inhibition that is independent of both opioid and cannabinoid systems.

## Discussion

Pain, a common symptom and disorder, presents a global public health challenge due to the symptom itself and adverse effects associated with current medications, such as opioids and cannabinoids. Despite their effectiveness, opioids are subject to scrutiny due to concerns such as tolerance, dependence, and respiratory depression (Stein, 2018). Meanwhile, endocannabinoids and cannabinoid receptors in mesocorticolimbic system have been identified as playing a crucial role in drug consumption and the development of drug addiction (Manzanares et al., 2018). The PVT serves as a critical neural hub for sensory processing in the brain, including pain sensation (Barson et al., 2020; Zhang et al., 2023; Zhu et al., 2016; Cui et al., 2024; Chang et al., 2019). Compelling evidence strongly implicates the PVT as a promising target for developing non-opioid analgesics (Zhang et al., 2023). Yet, the underlying molecular mechanisms regulating non-opioid analgesia and pain perception remain incompletely understood. Our data demonstrated that, in the PVT, A_2A_R are predominantly expressed in Iba1-positive microglia cells, and their expression remained unchanged in mice with CFA-induced persistent pain. Intra-PVT infusion of an A_2A_R agonist induced pain-like behavior in mice, whereas infusion of A_2A_R antagonists mitigated CFA-induced pain behaviors. Importantly, antagonizing PVT local A_2A_R induced analgesic effects cannot be prevented by pretreatment with naloxone or rimonabant, inhibitors of opioid and cannabinoid receptors CB1R, respectively. The results suggest a novel functional role for PVT A_2A_R in pain sensation and analgesia, thereby providing a molecular target for future analgesic development.

Recent research has uncovered the pivotal role of the PVT in the development and maintenance of pain, mainly mediated through its connections with downstream brain regions such as the basolateral amygdala, the central amygdala, the medial prefrontal cortex, and the NAc (Zhang et al., 2023; Cui et al., 2024; Tang et al., 2023; Liang et al., 2020). PVT glutamatergic neurons exhibited higher excitability in response to nociceptive stimuli and under various pain states, subsequently activating their downstream targets (Tang et al., 2023; Liang et al., 2020; Cheng et al., 2017). Concurrently, inhibiting PVT activity with chemogenetic and optogenetic approaches has been shown to alleviate pain (Zhang et al., 2023; Cui et al., 2024), similar to the effect observed in healthy volunteers treated with opioids (Eckhardt et al., 2000). Numerous studies have also focused on modulating neuronal factors to reduce excessive PVT activity as a strategy for mitigating pain or pain-related affective disorders (Zhang et al., 2023; Cui et al., 2024; Deng et al., 2023; Zeng et al., 2021a; Hou et al., 2023). This body of evidence indicates that the PVT serves as an important hub in regulating pain sensation and analgesia. Furthermore, molecular studies have identified potential targets underlying the pain regulating effects of the PVT neurons, including orexin receptor, dopamine receptor 3 and melanocortin 4 receptor (Zhang et al., 2023; Zeng et al., 2021b; Korczeniewska et al., 2021). Here, we further demonstrated an essential role of microglial A_2A_R within the PVT in pain regulation and analgesia independent opioid and cannabinoid receptors, providing a novel avenue for future development of analgesic strategies. Additionally, our results also indicate an essential role of PVT microglia in regulating pain sensation and analgesia. However, the precise mechanisms by which microglial A_2A_R exert their functional roles remain unclear.

In a mouse model resembling trigeminal neuralgia, elevated extracellular ATP/adenosine levels were observed in the ventral hippocampus CA1 area, accompanied by robust activation of astrocytes and microglia (Lv et al., 2024). Conversely, ATL313, an A_2A_R agonist, reduced nerve injury-induced microglial activation in the spinal cord and exerted sustained relief from allodynia across multiple nerve injury models. This effect was associated with reduced protein kinase A and protein kinase C, as well as TNFα production by microglia and astrocytes (Loram et al., 2009; Kwilasz et al., 2018; Loram et al., 2013). These evidence indicated a promising role of A_2A_R in pain sensation, possibly via its neuro-protective role in suppressing microglial activation and inflammatory responses (Franco et al., 2021). Furthermore, they offer insights into the mechanisms underlying the pain-regulating actions of PVT microglial A_2A_ receptors. And future mechanical studies are needed to identify how PVT microglial A_2A_R regulates pain sensation and analgesia, particularly focusing on maladaptation in microglia activation, microglia-neuron interaction and local adenosine release as possible candidate directions for investigation.

Adenosine is a natural purinergic nucleoside crucial for regulating various physiological processes, including cardiovascular function, immune response, sleep patterns, and pain modulation (Jacobson and Gao, 2006). It acts by binding to four G protein-coupled receptors: A_1_, A_2A_, A_2B_, and A_3_ adenosine receptors, each binding to distinct G protein subunits (Pasquini et al., 2021). A_1A_R and A_3A_R primarily activate Gi proteins, leading to potassium channel stimulation, inhibition of calcium channels, decreased cyclic adenosine monophosphate (cAMP) production, and attenuation of glial activation and inflammatory mediator release, thereby exhibiting mainly anti-nociceptive effects (Sawynok, 2016). Conversely, A_2A_R and A_2B_R activation has been controversial due to their dual pro- and anti-nociceptive effects. Studies using A_2A_R knockout mice have shown attenuated acute and nerve injury-induced pain responses (Hussey et al., 2010). Intrathecal administration of A_2A_R agonists has demonstrated anti-allodynic effects in models of chronic constriction injury and spinal cord injury (Loram et al., 2009; Kwilasz et al., 2018). In rats with spared nerve injury, local administration of A_2A_R agonists in the affected sciatic nerve area alleviated thermal hyperalgesia through upregulated cAMP levels and phosphorylation of cAMP-response element binding protein (Joo et al., 2023). However, our findings indicate that intra-PVT infusion of an A_2A_R agonist induces allodynia, while an A_2A_R antagonist exhibits analgesic effects in both normal and pain-induced mice. Consistent with our results, activation of A_2A_R and A_2B_R in neuropathic pain models has been reported to induce pain hypersensitivity (Abo-Salem et al., 2004; Bura et al., 2008). A_2A_R-deficient mice display reduced mechanical allodynia and diminished thermal hyperalgesia induced by sciatic nerve injury (Bura et al., 2008). Furthermore, systemic administration of selective A_2B_R antagonist has shown antinociceptive effects in hot-plate tests (Abo-Salem et al., 2004). Blockade of A_2A_R with SCH58261, administered systemically or intrathecally, effectively counteracts nociception (Marcondes Sari et al., 2014; Sawynok and Reid, 2012). These variations might partly due to the differences in the type of nerve injury or sensitized physiological states (Sawynok, 2016), along with variations in the routes of drug administration. The specific adenosine receptors subtypes expressed in the PVT and their functional roles in regulating pain sensation and analgesia are valuable scientific questions deserved future investigations, which is a potential limitation of our present study.

In conclusion, our study has demonstrated an essential role PVT microglial A_2A_R in pain sensation and analgesia. Of note, the analgesic property induced by antagonizing PVT local A_2A_R cannot be prevented by systematically administration of antagonists of opioid or cannabinoid receptors. This suggests a novel analgesic mechanism which could be used for developing safer and non-addictive analgesics.

## Data Availability

The raw data supporting the conclusions of this article will be made available by the authors, without undue reservation.

## Supplementary material

SUPPLEMENTARY FIGURE 1 Original western blot images supplemented to Figure 2E. **(A)** Original western blots of A_2A_R protein expression in mice received saline and CFA treatments. (Sham, *n* = 6 mice; 4 h, *n* = 6 mice; 3 days, *n* = 6 mice). **(B)** Original western blots of GAPDH expression in mice received saline and CFA treatments. (Sham, *n* = 6 mice; 4 h, *n* = 6 mice; 3 days, *n* = 6 mice).

SUPPLEMENTARY FIGURE 2 Distance traveled of SCH58261-treated mice and Vehicle-treated mice. (Vehicle, n = 8 mice; SCH58261, n = 7 mice). **p* < 0.05. Data analyzed with unpaired t test.

SUPPLEMENTARY TABLE 1 Extended statistical information for Figures 2–6.
