# Supplementary material for: Microglial adenosine A2A receptor in the paraventricular thalamic nucleus regulates pain sensation and analgesic effects independent of opioid and cannabinoid receptors
Source: Front Pharmacol. 2024 Dec 19;15:1467305. doi: 10.3389/fphar.2024.1467305 (PMC11693661; doi:10.3389/fphar.2024.1467305)
Supplement: Supplementary file 1 [file Table1.docx]

**Supplementary Table 1. Extended statistical information for Fig. 2-6.**

| **Figure** | **n mice/group, mean ± SEM or median (P25, P75)** | **Primary statistic** | ***Post-hoc* test** | **Comparison** | ***p* value** | **Notation** | ***F/t* statistic** |
| --- | --- | --- | --- | --- | --- | --- | --- |
| Fig. 2C | Sham, n = 20, 0.6310 ± 0.0551 g  4h, n = 20, 0.1685 ± 0.0293 g  3d, n = 20, 0.1665 ± 0.0260 g | One-way ANOVA | Tukey's | Main effect of group | <0.0001 | *** | F (2, 57) = 50.19 |
|  |  |  |  | Sham vs. 4h | <0.0001 | *** |  |
|  |  |  |  | Sham vs. 3d | <0.0001 | *** |  |
|  |  |  |  | 4h vs. 3d | 0.9993 | ns |  |
| Fig. 2D | Sham, n = 20, 12.49 ± 0.5601 s  4h, n = 20, 5.443 ± 0.3217 s  3d, n = 20, 5.910 ± 0.3626 s | One-way ANOVA | Tukey's | Main effect of group | <0.0001 | *** | F (2, 57) = 84.89 |
|  |  |  |  | Sham vs. 4h | <0.0001 | *** |  |
|  |  |  |  | Sham vs. 3d | <0.0001 | *** |  |
|  |  |  |  | 4h vs. 3d | 0.7212 | ns |  |
| Fig. 2F | Sham, n = 6, 1.000 ± 0.1079  4h, n = 6, 0.9160 ± 0.0836  3d, n = 6, 0.9924 ± 0.0607 | One-way ANOVA | Tukey's | Main effect of group | 0.7734 | ns | F (2, 15) = 0.2905 |
|  |  |  |  | Sham vs. 4h | 0.9979 | ns |  |
|  |  |  |  | Sham vs. 3d | 0.8078 | ns |  |
| Fig. 3C | Vehicle, n = 11, 0.7182 ± 0.0990 g  CGS21680, n = 11, 0.3372 ± 0.0702 g | Unpaired t test |  |  | 0.0052 | ** | t (20) = 3.139 |
| Fig. 3D | Vehicle, n = 11, 12.22 ± 1.064 s  CGS21680, n = 11, 9.273 ± 0.8958 s | Unpaired t test |  |  | 0.0465 | * | t (20) = 2.123 |
| Fig. 3E | Vehicle, n = 7, 0.6100 ± 0.0766 g  SCH58261 0.4ng, n = 7, 0.6214 ± 0.1368 g  SCH58261 4ng, n = 7, 0.9957 ± 0.1562 g  SCH58261 40ng, n = 7, 1.140 ± 0.1408 g | One-way ANOVA | Tukey's | Main effect of group | 0.0165 | * | F (3, 24) = 4.163 |
|  |  |  |  | Vehicle vs. SCH58261 0.4ng | >0.9999 | ns |  |
|  |  |  |  | Vehicle vs. SCH58261 4ng | 0.1885 | ns |  |
|  |  |  |  | Vehicle vs. SCH58261 40ng | 0.0405 | * |  |
|  |  |  |  | SCH58261 0.4ng vs. SCH58261 4ng | 0.2096 | ns |  |
|  |  |  |  | SCH58261 0.4ng vs. SCH58261 40ng | 0.0462 | * |  |
|  |  |  |  | SCH58261 4ng vs. SCH58261 40ng | 0.8637 | ns |  |
| Fig. 3F | Vehicle, n = 7, 11.00 ± 1.020 s  SCH58261 0.4ng, n = 7, 10.64 ± 0.9670 s  SCH58261 4ng, n = 7, 13.44 ± 1.229 s  SCH58261 40ng, n = 7, 15.22 ± 0.9770 s | One-way ANOVA | Tukey's | Main effect of group | 0.0161 | * | F (3, 24) = 4.192 |
|  |  |  |  | Vehicle vs. SCH58261 0.4ng | 0.9950 | ns |  |
|  |  |  |  | Vehicle vs. SCH58261 4ng | 0.3766 | ns |  |
|  |  |  |  | Vehicle vs. SCH58261 40ng | 0.0427 | * |  |
|  |  |  |  | SCH58261 0.4ng vs. SCH58261 4ng | 0.2633 | ns |  |
|  |  |  |  | SCH58261 0.4ng vs. SCH58261 40ng | 0.0251 | * |  |
|  |  |  |  | SCH58261 4ng vs. SCH58261 40ng | 0.6372 | ns |  |
|  |  |  |  |  |  |  |  |
| Fig. S2 | Vehicle, n = 8, 17.39 ± 0.8162 g  SCH, n = 7, 21.00 ± 1.13 g | Unpaired t test |  |  | 0.0206 | * | t(13) = 2.634 |
| Fig. 4B | Sham Vehicle, n = 12, 0.5275 ± 0.0417 g  Sham SCH, n = 11, 0.7873 ± 0.0665 g  CFA_4h Vehicle, n = 15, 0.1860 ± 0.0220 g  CFA_4h SCH, n = 16, 0.5288 ± 0.0711 g  CFA_3d Vehicle, n = 10, 0.2960 ± 0.0515 g  CFA_3d SCH, n = 8, 0.7800 ± 0.0616 g | One-way ANOVA | Tukey's | Main effect of group | <0.0001 | *** | F (5, 66) = 18.33 |
|  |  |  |  | Sham Vehicle vs. Sham SCH | 0.0241 | * |  |
|  |  |  |  | Sham Vehicle vs. CFA_4h Vehicle | 0.0003 | *** |  |
|  |  |  |  | Sham Vehicle vs. CFA_4h SCH | >0.9999 | ns |  |
|  |  |  |  | Sham Vehicle vs. CFA_3d Vehicle | 0.0721 | ns |  |
|  |  |  |  | Sham Vehicle vs. CFA_3d SCH | 0.0615 | ns |  |
|  |  |  |  | Sham SCH vs. CFA_4h Vehicle | <0.0001 | *** |  |
|  |  |  |  | Sham SCH vs. CFA_4h SCH | 0.0139 | * |  |
|  |  |  |  | Sham SCH vs. CFA_3d Vehicle | <0.0001 | *** |  |
|  |  |  |  | Sham SCH vs. CFA_3d SCH | >0.9999 | ns |  |
|  |  |  |  | CFA_4h Vehicle vs. CFA_4h SCH | <0.0001 | *** |  |
|  |  |  |  | CFA_4h Vehicle vs. CFA_3d Vehicle | 0.7332 | ns |  |
|  |  |  |  | CFA_4h Vehicle vs. CFA_3d SCH | <0.0001 | *** |  |
|  |  |  |  | CFA_4h SCH vs. CFA_3d Vehicle | 0.0449 | * |  |
|  |  |  |  | CFA_4h SCH vs. CFA_3d SCH | 0.0432 | * |  |
|  |  |  |  | CFA_3d Vehicle vs. CFA_3d SCH | <0.0001 | *** |  |
| Fig. 4C | Sham Vehicle, n = 12, 11.88 ± 0.7138 s  Sham SCH, n = 11, 15.51 ± 0.6949 s  CFA_4h Vehicle, n = 15, 5.252 ±0.4492 s  CFA_4h SCH, n = 16, 8.803 ± 0.9150 s  CFA_3d Vehicle, n = 10, 6.615 ± 0.7234 s  CFA_3d SCH, n = 8, 12.82 ± 0.8236 s | One-way ANOVA | Tukey's | Main effect of group | <0.0001 | *** | F (5, 66) = 26.78 |
|  |  |  |  | Sham Vehicle vs. Sham SCH | 0.0172 | * |  |
|  |  |  |  | Sham Vehicle vs. CFA_4h Vehicle | <0.0001 | *** |  |
|  |  |  |  | Sham Vehicle vs. CFA_4h SCH | 0.0329 | * |  |
|  |  |  |  | Sham Vehicle vs. CFA_3d Vehicle | 0.0002 | *** |  |
|  |  |  |  | Sham Vehicle vs. CFA_3d SCH | 0.9695 | ns |  |
|  |  |  |  | Sham SCH vs. CFA_4h Vehicle | <0.0001 | *** |  |
|  |  |  |  | Sham SCH vs. CFA_4h SCH | <0.0001 | *** |  |
|  |  |  |  | Sham SCH vs. CFA_3d Vehicle | <0.0001 | *** |  |
|  |  |  |  | Sham SCH vs. CFA_3d SCH | 0.2423 | ns |  |
|  |  |  |  | CFA_4h Vehicle vs. CFA_4h SCH | 0.0043 | ** |  |
|  |  |  |  | CFA_4h Vehicle vs. CFA_3d Vehicle | 0.7946 | ns |  |
|  |  |  |  | CFA_4h Vehicle vs. CFA_3d SCH | <0.0001 | *** |  |
|  |  |  |  | CFA_4h SCH vs. CFA_3d Vehicle | 0.3098 | ns |  |
|  |  |  |  | CFA_4h SCH vs. CFA_3d SCH | 0.0088 | ** |  |
|  |  |  |  | CFA_3d Vehicle vs. CFA_3d SCH | <0.0001 | *** |  |
| Fig. 5B | Saline + Saline, n = 8, 1.026 ± 0.1040 g  Saline + Morphine, n = 8, 1.786 ± 0.3367 g  Naloxone + Saline, n = 8, 1.104 ± 0.1168 g  Naloxone + Morphine, n = 8, 0.8875 ± 0.1077 g | One-way ANOVA | Tukey's | Main effect of group | 0.0030 | ** | F (3, 28) = 5.896 |
|  |  |  |  | Saline+Saline vs. Naloxone+Saline | 0.8554 | ns |  |
|  |  |  |  | Saline+Saline vs. Saline+Morphine | 0.0136 | * |  |
|  |  |  |  | Saline+Saline vs. Naloxone+Morphine | 0.8554 | ns |  |
|  |  |  |  | Naloxone+Saline vs. Saline+Morphine | 0.0190 | * |  |
|  |  |  |  | Naloxone+Saline vs. Naloxone+Morphine | 0.8554 | ns |  |
|  |  |  |  | Saline+Morphine vs. Naloxone+Morphine | 0.0044 | ** |  |
| Fig. 5C | Saline + Saline, n = 8, 9.069 ± 0.5830 s  Saline + Morphine, n = 8, 11.99 ± 0.7417 s  Naloxone + Saline, n = 8, 8.341 ± 0.5302 s  Naloxone + Morphine, n = 8, 7.555 ± 1.050 s | One-way ANOVA | Tukey's | Main effect of group | 0.0017 | ** | F (3, 28) = 6.575 |
|  |  |  |  | Saline + Saline vs. Saline + Morphine | 0.0495 | * |  |
|  |  |  |  | Saline + Saline vs. Naloxone + Saline | 0.9029 | ns |  |
|  |  |  |  | Saline + Saline vs. Naloxone + Morphine | 0.4980 | ns |  |
|  |  |  |  | Saline + Morphine vs. Naloxone + Saline | 0.0099 | ** |  |
|  |  |  |  | Saline + Morphine vs. Naloxone + Morphine | 0.0015 | ** |  |
|  |  |  |  | Naloxone + Saline vs. Naloxone + Morphine | 0.8812 | ns |  |
| Fig. 5D | Saline + Saline, n = 8, 0.35 (0.25, 0.6075) g  Naloxone + Saline, n = 8, 0.38 (0.2725, 0.7025) g  Saline + Morphine, n = 8, 1.07 (0.5425, 1.30) g  Naloxone + Morphine, n = 8, 0.295 (0.22, 0.4525)g | One-way ANOVA | Kruskal-Wallis test | Main effect of group | 0.0018 | ** | H = 15.04 |
|  |  |  |  | Saline+Saline vs. Naloxone+Saline | >0.9999 | ns |  |
|  |  |  |  | Saline+Saline vs. Saline+Morphine | 0.0387 | * |  |
|  |  |  |  | Saline+Saline vs. Naloxone+Morphine | >0.9999 | ns |  |
|  |  |  |  | Naloxone+Saline vs. Saline+Morphine | 0.0844 | ns |  |
|  |  |  |  | Naloxone+Saline vs. Naloxone+Morphine | >0.9999 | ns |  |
|  |  |  |  | Saline+Morphine vs. Naloxone+Morphine | 0.0011 | ** |  |
| Fig. 5E | Saline + Saline, n = 8, 6.796 ± 0.7240 s  Saline + Morphine, n = 8, 10.52 ± 0.8522 s  Naloxone + Saline, n = 8, 6.931 ±0.7023 s  Naloxone + Morphine, n = 8, 5.663 ± 0.1469 s | One-way ANOVA | Tukey's | Main effect of group | 0.0001 | *** | F (3, 28) = 10.06 |
|  |  |  |  | Saline + Saline vs. Saline + Morphine | 0.0025 | ** |  |
|  |  |  |  | Saline + Saline vs. Naloxone + Saline | 0.9989 | ns |  |
|  |  |  |  | Saline + Saline vs. Naloxone + Morphine | 0.6278 | ns |  |
|  |  |  |  | Saline + Morphine vs. Naloxone + Saline | 0.0036 | ** |  |
|  |  |  |  | Saline+Morphine vs. Naloxone+Morphine | <0.0001 | *** |  |
|  |  |  |  | Naloxone + Saline vs. Naloxone + Morphine | 0.5397 | ns |  |
| Fig. 6B | Vehicle + Saline, n = 11, 0.65 (0.52, 0.65) g  Vehicle + Naloxone, n = 11, 0.65 (0.41, 0.65) g  SCH + Saline, n = 11, 0.98 (0.98, 1.30) g  SCH + Naloxone, n = 11, 0.98 (0.87, 1.43) g | One-way ANOVA | Kruskal-Wallis test | Main effect of group | <0.0001 | *** | H = 28.55 |
|  |  |  |  | Vehicle+Saline vs. Vehicle+Naloxone | >0.9999 | ns |  |
|  |  |  |  | Vehicle+Saline vs. SCH+Saline | 0.0011 | ** |  |
|  |  |  |  | Vehicle+Saline vs. SCH+Naloxone | 0.0044 | ** |  |
|  |  |  |  | Vehicle+Naloxone vs. SCH+Saline | 0.0002 | *** |  |
|  |  |  |  | Vehicle+Naloxone vs. SCH+Naloxone | 0.0009 | *** |  |
|  |  |  |  | SCH+Saline vs. SCH+Naloxone | >0.9999 | ns |  |
| Fig. 6C | Vehicle + Saline, n = 11, 12.28 ± 0.4765 s  Vehicle + Naloxone, n = 11, 11.76 ± 0.7924 s  SCH + Saline, n = 11, 12.58 ± 0.5304 s  SCH + Naloxone, n = 11, 11.14 ± 0.6761 s | One-way ANOVA | Tukey's | Main effect of group | 0.0004 | *** | F (3, 40) = 7.603 |
|  |  |  |  | Vehicle + Saline vs. Vehicle + Naloxone | 0.9361 | ns |  |
|  |  |  |  | Vehicle + Saline vs. SCH + Saline | 0.0083 | ** |  |
|  |  |  |  | Vehicle + Saline vs. SCH + Naloxone | 0.0539 | ns |  |
|  |  |  |  | Vehicle + Naloxone vs. SCH + Saline | 0.0016 | ** |  |
|  |  |  |  | Vehicle + Naloxone vs. SCH + Naloxone | 0.0126 | * |  |
|  |  |  |  | SCH + Saline vs. SCH + Naloxone | 0.8802 | ns |  |
| Fig. 6D | Vehicle + Saline, n = 10, 0.3150 (0.1525, 0.41) g  Vehicle + Naloxone, n = 9, 0.22 (0.155, 0.34) g  SCH + Saline, n = 8, 0.705 (0.65, 0.9525) g  SCH + Naloxone, n = 12, 0.57 (0.4125, 0.6475) g | One-way ANOVA | Kruskal-Wallis test | Main effect of group | <0.0001 | *** | H = 25.67 |
|  |  |  |  | Vehicle + Saline vs. Vehicle + Naloxone | >0.9999 | ns |  |
|  |  |  |  | Vehicle + Saline vs. SCH + Saline | 0.0006 | *** |  |
|  |  |  |  | Vehicle + Saline vs. SCH + Naloxone | 0.0463 | * |  |
|  |  |  |  | Vehicle + Naloxone vs. SCH + Saline | 0.0001 | *** |  |
|  |  |  |  | Vehicle + Naloxone vs. SCH + Naloxone | 0.0100 | * |  |
|  |  |  |  | SCH + Saline vs. SCH + Naloxone | 0.7474 | ns |  |
| Fig. 6E | Vehicle + Saline, n = 10, 6.615 ± 0.7234 s  Vehicle + Naloxone, n = 9, 5.160 ± 0.3638 s  SCH + Saline, n = 8, 12.82 ± 0.8236 s  SCH + Naloxone, n = 12, 13.25 ± 1.097 s | One-way ANOVA | Tukey's | Main effect of group | <0.0001 | *** | F (3, 35) = 23.43 |
|  |  |  |  | Vehicle + Saline vs. Vehicle + Naloxone | 0.6452 | ns |  |
|  |  |  |  | Vehicle + Saline vs. SCH + Saline | 0.0001 | *** |  |
|  |  |  |  | Vehicle + Saline vs. SCH + Naloxone | <0.0001 | *** |  |
|  |  |  |  | Vehicle + Naloxone vs. SCH + Saline | <0.0001 | *** |  |
|  |  |  |  | Vehicle + Naloxone vs. SCH + Naloxone | <0.0001 | *** |  |
|  |  |  |  | SCH + Saline vs. SCH + Naloxone | 0.9850 | ns |  |
| Fig. 7B | Vehicle + Saline, n = 11, 0.65 (0.52, 0.65) g  Vehicle + Rimonabant, n = 11, 0.65 (0.49, 0.65) g  SCH + Saline, n = 11, 0.98 (0.98, 1.30) g  SCH + Rimonabant, n = 11, 1.3 (0.998, 1.75) g | One-way ANOVA | Kruskal-Wallis test | Main effect of group | <0.0001 | *** | H = 25.12 |
|  |  |  |  | Vehicle +Saline vs. Vehicle + Rimonabant | >0.9999 | ns |  |
|  |  |  |  | Vehicle + Saline vs. SCH + Saline | 0.0069 | ** |  |
|  |  |  |  | Vehicle + Saline vs. SCH + Rimonabant | 0.0006 | *** |  |
|  |  |  |  | Vehicle + Rimonabant vs. SCH + Saline | 0.0104 | * |  |
|  |  |  |  | Vehicle+Rimonabant vs. SCH+Rimonabant | 0.0010 | *** |  |
|  |  |  |  | SCH + Saline vs. SCH + Rimonabant | >0.9999 | ns |  |
| Fig. 7C | Vehicle + Saline, n = 11, 11.72 ± 0.6236 s  Vehicle + Rimonabant, n = 11, 11.47 ± 0.3696 s  SCH + Saline, n = 11, 14.42 ± 0.9108 s  SCH + Rimonabant, n = 11, 15.78 ± 0.6239 s | One-way ANOVA | Tukey's | Main effect of group | <0.0001 | *** | F (3, 40) = 10.14 |
|  |  |  |  | Vehicle+ Saline vs. Vehicle + Rimonabant | >0.9999 | ns |  |
|  |  |  |  | Vehicle + Saline vs. SCH + Saline | 0.0361 | * |  |
|  |  |  |  | Vehicle + Saline vs. SCH + Rimonabant | 0.0005 | *** |  |
|  |  |  |  | Vehicle + Rimonabant vs. SCH + Saline | 0.0180 | * |  |
|  |  |  |  | Vehicle+Rimonabant vs. SCH+Rimonabant | 0.0002 | *** |  |
|  |  |  |  | SCH + Saline vs. SCH + Rimonabant | 0.6290 | ns |  |
| Fig. 7D | Vehicle + Saline, n = 8, 0.1563 ± 0.0379 g  Vehicle + Rimonabant, n = 8, 0.1463 ± 0.0206 g  SCH + Saline, n = 8, 0.5138 ± 0.0781 g  SCH + Rimonabant, n = 7, 0.4886 ± 0.0963 g | One-way ANOVA | Tukey's | Main effect of group | 0.0001 | *** | F (3, 27) = 10.29 |
|  |  |  |  | Vehicle +Saline vs. Vehicle + Rimonabant | 0.9995 | ns |  |
|  |  |  |  | Vehicle + Saline vs. SCH + Saline | 0.0020 | ** |  |
|  |  |  |  | Vehicle + Saline vs. SCH + Rimonabant | 0.0057 | ** |  |
|  |  |  |  | Vehicle + Rimonabant vs. SCH + Saline | 0.0015 | ** |  |
|  |  |  |  | Vehicle+Rimonabant vs. SCH+Rimonabant | 0.0044 | ** |  |
|  |  |  |  | SCH + Saline vs. SCH + Rimonabant | 0.9924 | ns |  |
| Fig. 7E | Vehicle + Saline, n = 8, 4.961 ± 0.3937 s  Vehicle + Rimonabant, n = 8, 5.861 ± 0.3894 s  SCH + Saline, n = 8, 7.859 ± 0.7944 s  SCH + Rimonabant, n = 7, 8.724 ± 0.5363 s | One-way ANOVA | Tukey's | Main effect of group | 0.0002 | *** | F (3, 27) = 9.611 |
|  |  |  |  | Vehicle+ Saline vs. Vehicle + Rimonabant | 0.6542 | ns |  |
|  |  |  |  | Vehicle + Saline vs. SCH + Saline | 0.0045 | ** |  |
|  |  |  |  | Vehicle + Saline vs. SCH + Rimonabant | 0.0004 | *** |  |
|  |  |  |  | Vehicle + Rimonabant vs. SCH + Saline | 0.0695 | ns |  |
|  |  |  |  | Vehicle+Rimonabant vs. SCH+Rimonabant | 0.0069 | ** |  |
|  |  |  |  | SCH + Saline vs. SCH + Rimonabant | 0.7037 | ns |  |
