# Supplementary figures and images for: Microglial adenosine A2A receptor in the paraventricular thalamic nucleus regulates pain sensation and analgesic effects independent of opioid and cannabinoid receptors
Source: Front Pharmacol. 2024 Dec 19;15:1467305. doi: 10.3389/fphar.2024.1467305 (PMC11693661; doi:10.3389/fphar.2024.1467305)

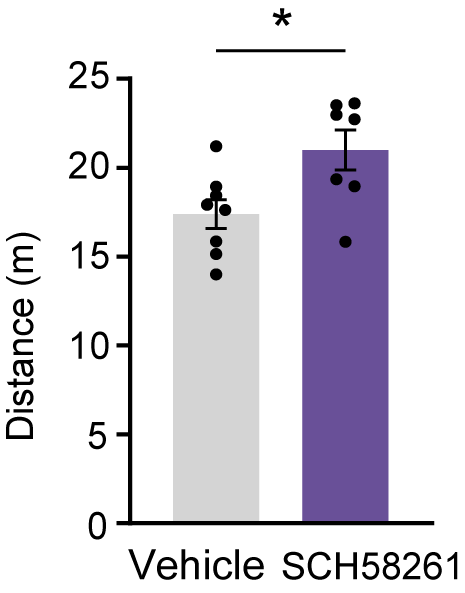

Supplement: Supplementary file 2 [file Image2.tif]

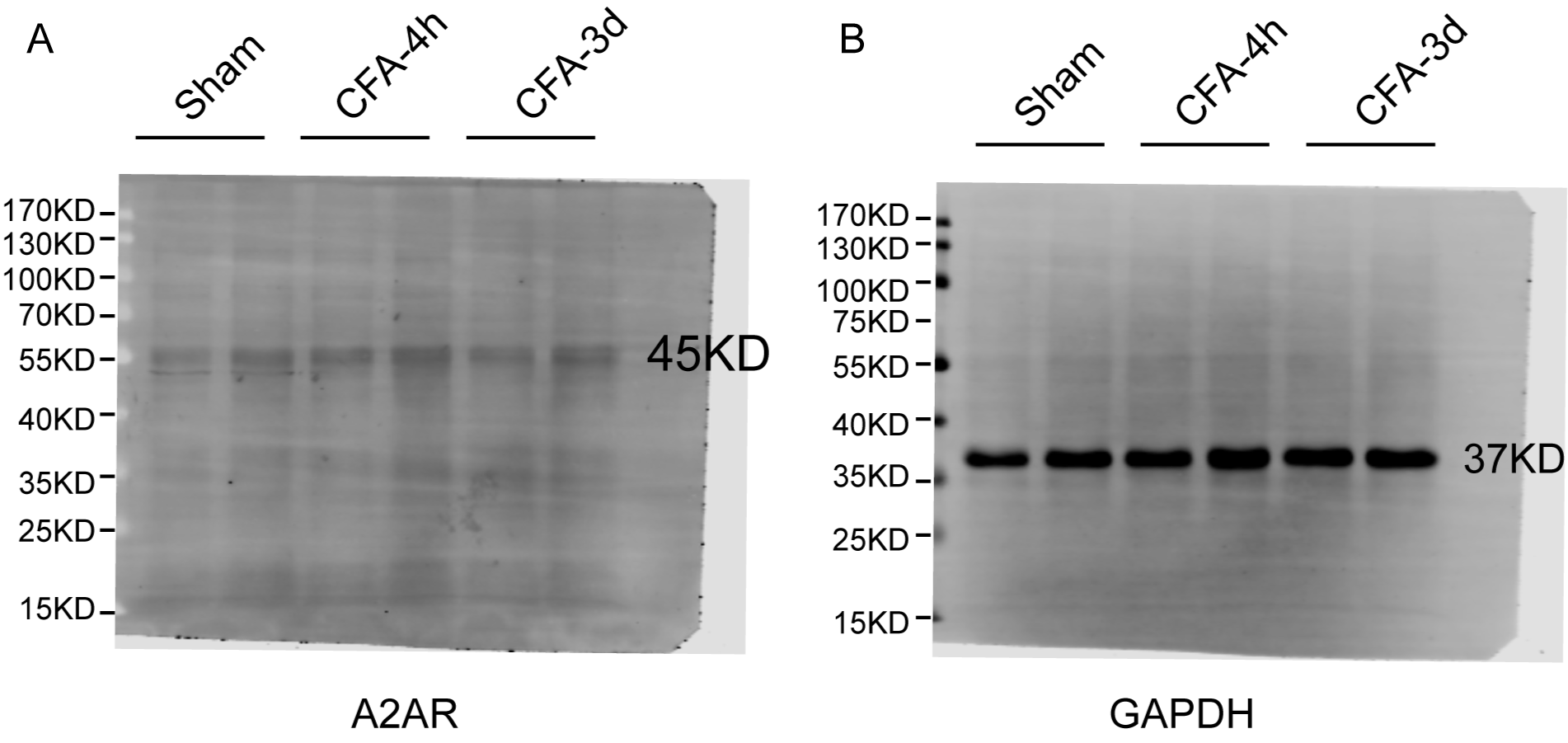

Supplement: Supplementary file 3 [file Image1.tif]
